# Editorial: Highlights in cognition: visual-spatial processing

**DOI:** 10.3389/fpsyg.2026.1780957

**Published:** 2026-01-22

**Authors:** Eirini Mavritsaki, Jessica Ann Diaz, Jipeng Qiang

**Affiliations:** 1 Department of Psychology, Birmingham City University, Birmingham, United Kingdom; 2 School of Information and Artificial Intelligence, Yangzhou University, Jiangsu, China

**Keywords:** attention, cognition, cultural differences, motor imagery, neuroimaging, visual-spatial processing

Visual-spatial processing is considered to be the core pillar of human cognition as it effects how we perceive, represent, manipulate, and act within space (Carrasco, 2018; Lecce et al.; Trés and Brucki, 2014). These processes are integrated across multiple cognitive domains including mental rotation and the embodiment of action plans to the visual foundation of reading and remote associative thinking. Visual-spatial processes are woven through perception (Trés and Brucki, 2014), memory (McAfoose and Baune, 2009), attention (Carrasco, 2018), and language (Gordon et al., 2018; Marton, 2008) rendering them essential to how we interact with and understand our environment. Furthermore, visual-spatial processing is significantly influenced by cultural differences (Gonthier, 2022). Cultural background shapes how individuals perceive spatial relationships, interpret visual information and solve spatial problems (Boduroglu and Shah, 2017; Gutchess and Rajaram, 2023; Mavritsaki et al., 2025; Nisbett et al., 2001). It is therefore essential to account for cross-cultural differences in research on visual-spatial processing.

For this Frontiers Research Topic, *Highlights in Cognition: Visual-spatial Processing*, our aim was to gather work that both exemplifies methodological breadth and advances theoretical understanding across developmental, neurocognitive, applied, and translational domains. The six contributions assembled here: experimental (Chen and Zhang; Gagné et al.; Lecce et al.; Saran and Marotta), computational (Ji et al.), developmental (Lecce et al.) and neuroimaging (Wu and Chen), collectively showcase how visual-spatial processing informs a wide variety of human behaviors and cognitive capacities, and they point to promising directions for future research.

A recurring theme across these articles is the multilevel character of visual-spatial processing, which operates in multiple interconnected levels of cognitive function. Visual-spatial processing is simultaneously perceptual, involving low level phenomena like masking and contrast interactions (Chen and Zhang), oculomotor and attentional as seen on reading and rapid automatised naming (Lecce et al.), motoric and embodied as presented in hand laterality judgments and motor imagery (Saran and Marotta), and conceptual as seen on visual-spatial and remote associations (Wu and Chen). Several contributions emphasize interactions among these levels, revealing that visual-spatial processes cannot be understood in isolation. For example, research shows how attentional demand modulates masking effects at the perceptual level (Ramachandran and Cobb, 1995), while biomechanical constraints shape laterality judgments at the motor level (Kosslyn et al., 2001; Vannuscorps et al., 2012). These findings highlight a crucial insight that visual-spatial functioning is neither a unitary construct nor an isolated cognitive module. Instead, it represents an integrated system where perceptual, attentional, motor, and conceptual processes interact and mutually influence one another. This multi-level approach has important implications for understanding individual differences, developmental trajectories, and clinical disorders that affect visual-spatial cognition.

Saran and Marotta's study on implicit motor imagery in the Hand Laterality Judgment Task addresses how aging affects the strategy people adopt when judging laterality. Their careful comparison of visual vs. motor strategies, combined with manipulations of realistic vs. mannequin hands and viewpoint, reveals both age-related slowing and qualitative shifts in error patterns. The finding that older adults retain sensitivity to biomechanical constraints (faster responses for medial orientations) while showing distinct error profiles highlights the nuanced ways that sensorimotor simulation and visual analysis interact across the lifespan. This work adds to literature on embodied cognition and aging, and suggests that task design (stimulus realism, viewpoint) can selectively engage motor vs. visual problem-solving routes.

Ji et al. take a computational and applied tack by linking visual-spatial skills to academic performance using deep learning. Their predictive classification system, framed as an expert system, demonstrates the utility of big data and AI for identifying how visual-spatial competencies contribute to success in science and engineering education. This contribution is important for two reasons: it underscores the practical value of measuring visual-spatial skills in educational contexts, and it exemplifies how cognitive constructs can be harnessed within predictive, personalized learning frameworks. The study invites future work that integrates psychometric precision with model interpretability and educational intervention design.

Light and its non-visual effects form the focus of Gagné et al., who experimentally manipulate melanopic stimulation to assess blue-enriched lighting on vigilance, visual performance, and subjective comfort. Their finding, namely reduced sleepiness yet mixed effects on visual comfort and contrast sensitivity, reminds us that optimizing environments for cognitive performance is a multidimensional problem. Practical applications (workplace lighting, classroom design, shift work) must balance alertness benefits with visual comfort and perceptual demands; this study provides a careful empirical basis for such trade-offs.

Developmental changes in reading-related visual-spatial and oculomotor skills are elegantly documented in Lecce et al.'s longitudinal Topological RAN study. By combining longitudinal measurement, error and self-correction metrics, and open analytic tools, the authors show that oculomotor control, attentional orientation, and executive aspects of reading evolve substantially during elementary school. Their emphasis on error patterns and self-correction, not just response times, helps paint a richer picture of reading acquisition, with implications for educational screening and remediation, especially for diverse and multilingual school populations.

Two psycholinguistic and neurocognitive studies probe processing at distinct representational levels. Chen and Zhang examine visual masking with Chinese characters, varying mask type, temporal sequence, and depth of character structure. The complex interactions they report, where task demands bias masking effects, highlight how attention and spatio-temporal dynamics determine whether visual information reaches higher lexical stages. Wu and Chen complement this approach by using fMRI to contrast visual-spatial and verbal remote association (CRRAT vs. CCRAT). Their neural dissociation, with distinct activations in frontal, parietal, and precuneal regions for visual-spatial associations, supports the view that remote associative thinking recruits modality-specific networks in addition to domain-general control systems.

Looking forward, several promising directions emerge. Greater integration across methods, combining behavioral paradigms with computational modeling and neuroimaging, will sharpen mechanistic accounts. Cross-linguistic and cross-cultural work (especially in reading and character processing) can reveal the generality of observed mechanisms. Finally, translational studies that evaluate interventions informed by basic visual-spatial science (training, environmental adjustments, educational tools) are needed to test causal leverage points for improving cognition and performance.

This Research Topic reflects the diversity of theoretical perspectives and methodological approaches that characterize research in visual-spatial cognition. It highlights how advances in experimental design, analytic methods, and applied modeling are collectively driving the field forward. We hope these articles inspire further interdisciplinary work that continues to map the rich terrain of visual-spatial processes and their central role in human thought and behavior.
